# Assessment and Documentation of Mental Capacity in Adult With Intellectual Disabilities Before Commencing Medications at Mid Mersey Care LD Services (1st of February – 1st of August 2025)

**DOI:** 10.1192/bjo.2026.11806

**Published:** 2026-06-30

**Authors:** Mahmoud Odeh, Shalini Gladston, Ashok Dinnahalli, Tasnim Elbadrawy, Nudrat Nudrat

**Affiliations:** Mersey Care, Liverpool, United Kingdom

## Abstract

**Aims:**

The Mental Capacity Act 2005 provides a statutory framework to empower and protect individuals who may lack the capacity to make specific decisions for themselves. Capacity assessments are essential to safeguarding the autonomy and rights of individuals, especially those with cognitive impairments, learning disabilities, or mental illness.

The rationale behind this audit is to ensure that capacity assessments are not only being completed when required, but that they are being carried out and clearly recorded in accordance with Mersey Care MC01 policy and the principles of the Mental Capacity Act (2005).

By identifying whether the assessment was done, and whether all steps were clearly recorded, this audit aims to promote compliance, improve clinical quality, and uphold patient rights across Mid Mersey Care LD services.

**Methods:**

This is a retrospective audit.

The inclusion criteria:

1. New referral to the team.

2. Adults aged 16 years or older lacking capacity / under the care of Intellectual Disabilities Community Mental Health team – Mid Mersey Care NHS Foundation Trust.

3. Cases where a significant decision was required (consent to treatment).

The exclusion criteria:

1. Not a new referral to the team.

2. Adults aged 16 years or older having capacity / NOT under the care of Intellectual Disabilities Community Mental Health team – Mid Mersey Care NHS Foundation Trust.

3. Cases where a significant decision was NOT required (consent to treatment).

The sample identified from an In-House Assessment Database and the data will be extracted from the electronic system (RIO).

Sample of 21 patient was identified.

**Results:**

Of the 21 cases audited, 18 had an assessment completed and 3 did not. Compliance level of 86%.

Of the 21 cases audited, 10 had clearly documented steps, 8 did not and 3 were N/A. Compliance level of 56%.

**Conclusion:**

While the Capacity Assessment was carried out and completed in the majority of cases. The clear documentation of all steps was only recorded in around half of them.

There is still a room for improvement by raising awareness during multidisciplinary team meeting and increase the completion of the existing capacity form on the electronic system and/or documentation of all capacity assessment steps during the patient review.

